# Feeling safe by means of remote cardiac rhythm surveillance after open cardiac surgery: a mixed method pilot feasibility study

**DOI:** 10.1186/s13019-026-03985-2

**Published:** 2026-04-09

**Authors:** Anna Nilsson, Jessica Åkesson, Marita Dalvindt, Henrik Bjursten, Anna Forsberg

**Affiliations:** 1 Department of Cardiothoracic Surgery, Lund University, Skåne University Hospital, Lund, Sweden; 2 Institute of Health Sciences, Lund University, Lund, S-222 40 Sweden

**Keywords:** Arrhythmias, Cardiac surgery, Cardiac nursing, Self-monitoring, Person-centred care, Feasibility

## Abstract

**Background:**

This is one of the few European studies of self-management and self-monitoring during the initial two weeks following discharge after open cardiac surgery. Typical outcomes are mortality, morbidity, surgical complications and additional medical treatment. Equally important for enabling person-centred care are the patients’ perspective and main concerns. The aim was to explore feasibility, self-management experiences and consequences in everyday life when self-monitoring with the Coala Heart Monitor^®^ after cardiac surgery.

**Methods:**

In this mixed method pilot feasibility study, we included 20 patients. One drop-out resulted in 12 men and 7 women with a mean age 59 years (range 27–82 years) participating in the study. Information about the participants’ heart rate was collected prospectively from discharge until two weeks post discharge. Retrospective interviews were performed after two weeks to explore their self-management experiences at home while performing self-monitoring. We used deductive content analysis in line with Hsieh and Shannon (2005), guided by Lorig & Holman’s (2003) self-management framework. Person centeredness was measured by the Being Taken Seriously Questionnaire. Approval was granted by the Swedish Ethical Review Authority (D.nr. 2020–05007).

**Results:**

The device was considered easy to use and administer regardless of the patient’s age. Self-monitoring supports self-management after open cardiac surgery by providing a sense of safety despite distance from the hospital. It stimulates learning about one’s body and symptoms in a highly useful way and can be adopted by both young and older patients, in addition to promoting relatives’ constructive participation. Self-monitoring also acts as a bridge between the discharged patient and healthcare professionals, thus strengthening and extending the caring relationship.

**Conclusion:**

In conclusion, self-monitoring provides tangible signs of well-being and control.

## Introduction

This novel mixed-method feasibility study was initiated during the Covid-19 pandemic and sought to explore and understand the possible benefits of combining patient engagement with remote surveillance and consultation after cardiac surgery. Approximately 1,100 cardiac operations are performed every year at our university hospital, of which the majority are Coronary Artery Bypass Grafts (CABG) or valve replacement procedures. People undergoing cardiac surgery experience many different symptoms postoperatively, related to both the procedure and subsequent medical treatment [1]. Some symptoms are non-cardiac specific, e.g. pain, fatigue and emotional distress, while others are heart related, e.g. dyspnoea, oesophageal dysphagia and nausea. One common postoperative complication in our unit is post-operative atrial fibrillation (POAF) with 30–40% of patients developing it on the post-surgical ward. Effective pharmacological treatment of POAF can be provided before discharge, facilitating good recovery at home. Postoperative symptoms after cardiac surgery might negatively affect the patients’ health related quality of life [1–2]. As such, effective treatment of POAF should be provided before discharge, facilitating better patient-related outcomes. As shown by Myles et al. [2] in their longitudinal follow-up of 120 adult patients undergoing cardiac surgery, deterioration during the first postoperative days predicted poor recovery three months later. Thus, exploring complications and symptoms as soon as possible after surgery is important for facilitating early interventions that might influence long-term recovery and prevent hospital re-admissions in times of pandemics or other crises. One way of exploration is the use of mobile applications which have been used to monitor symptoms in patients with chronic heart failure (CHF). According to Lefler et al. [3], self-management improved as patients with CHF became more aware of their symptoms. Self-monitoring seemed to increase patients’ self-confidence and interest in self-care [3–4]. Research about self-monitoring in persons with type 1-diabetes has been reviewed in a meta-analysis by Janapala et al. [5], which concluded that continuous blood glucose monitoring by healthcare professionals (HCPs) lowered HbA1c to a greater extent than self-monitoring. However, self-monitoring of blood glucose in combination with telephone follow up reduced HbA1c more than without such self-monitoring [6]. Several studies regarding the effect of self-monitoring on hypertension showed that it leads to lower blood pressure compared to conventional care [7–9]. There is one feasibility study from Australia involving self-monitoring after cardiac surgery [10]. Patients who had undergone cardiac surgery and suffered from POAF were asked to self-monitor with an electrocardiogram (ECG) four times a day as well as when having symptoms during the first four weeks after discharge. A total of 24% (*n* = 42) had at least one POAF episode during the study period [10]. According to the extensive literature review by Lee et al. [11] regarding virtual healthcare solutions, mobile technologies offer an opportunity to improve both the quality and utilization of cardiac rehabilitation.

After discharge from the thoracic ward patients are expected to recover at home for 6–8 weeks before visiting the outpatient clinic, i.e. self-management. During this almost two-month period no organized support or follow-up is provided at our hospital. Since our knowledge about this initial phase of recovery regarding symptom burden, various self-management challenges and prevalence of POAF is very limited it became the rationale behind this study. The aim was to explore feasibility, self-management experiences and consequences in everyday life when self-monitoring with the Coala Heart Monitor^®^ after cardiac surgery. In terms of feasibility we were interested in acceptability, adherence to the monitoring and technical performance. The primary research questions were: is self-monitoring a benefit or a burden while awaiting out-patient follow-up? How does self-monitoring with the Coala Heart Monitor^®^ after cardiac surgery affect the patients’ symptom management, body perception and possible postoperative distress? How do patients experience person-centeredness when self-monitoring after cardiac surgery? and What is the prevalence of POAF requiring treatment during the first weeks after cardiac surgery?

## Methods

### Research design

A convergent parallel mixed-methods study inspired by the design presented by Younas, Fàbregues, and Creswell [12]. Qualitative and quantitative inferences were combined and integrated in a meta-inference [12]. Meta-inferences involve the process of intensifying the use of the data to gain a deeper and more accurate understanding. To draw conclusions when generating meta-inferences, the qualitative and quantitative findings were compared, adding a synergetic effect and revealing valuable knowledge about the experiences of self-monitoring after cardiac surgery. The qualitative data was analysed first and then the quantitative data was added to the understanding of the overall findings. We have followed the SRQR standards [13] for presenting qualitative studies, as such studies constitute the main part of this mixed-method approach.

### Researcher characteristics and reflexivity

The researchers constantly reflected on their pre-understanding based on many years of professional work in the thoracic surgical unit as clinical nurse specialists, a thoracic surgeon and a professor in nursing.

### Context

A thoracic clinic in a large university hospital that performs more than 1,100 cardiac surgery procedures each year in addition to heart and lung transplantation.

### Sampling strategy and selection of participants

**Fig. 1:**
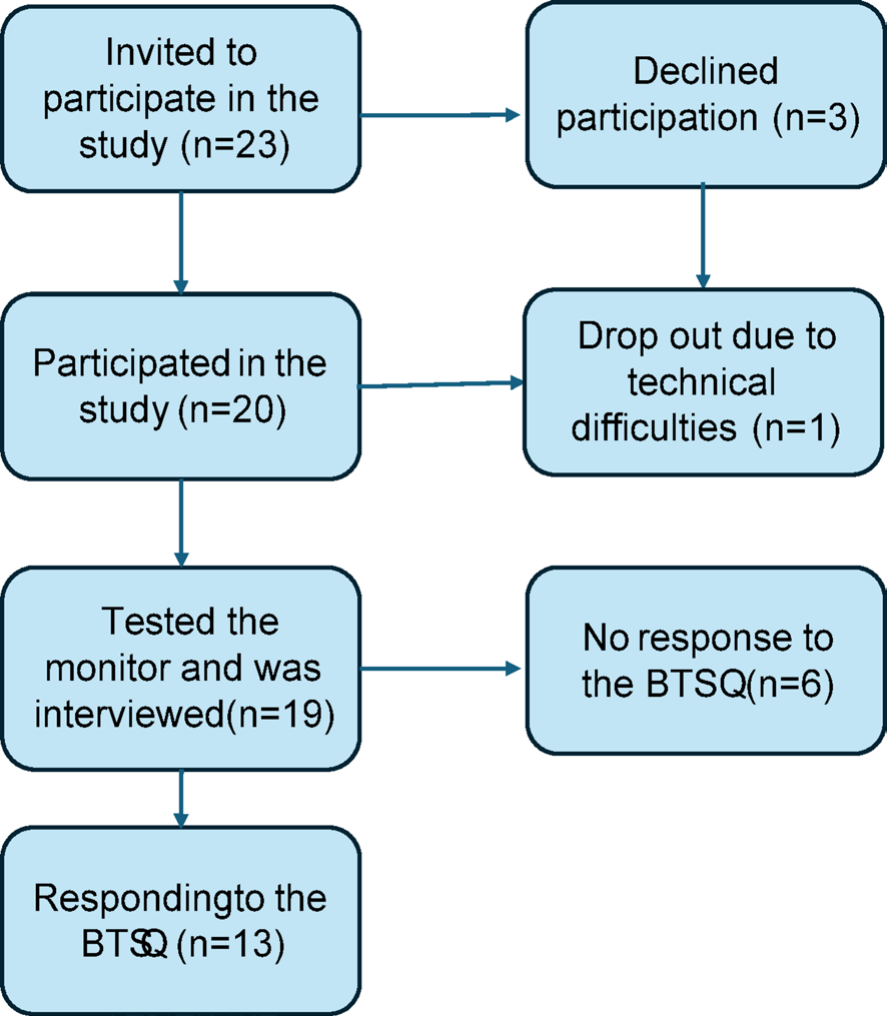
Overview of the inclusion process and final responders

Inclusion criteria were adults (> 18 years old), undergoing CABG or valve surgery, ability to provide informed consent, able to use the Coala Heart Monitor^®^ and providing written informed consent. Exclusion criteria were undergoing aortic surgery, acute lifesaving operations, being heart- or lung transplanted, Transcatheter Aortic Valve Implantation, Mechanical Circulatory Support, cognitively impaired or impaired fine motor ability preventing the proper use of the Coala Heart Monitor^®^. We used purposive sampling according to the flow chart in Fig. 1.

In total, 23 patients were consecutively invited from the study start when the day of their discharge was decided. We used the Swedish version of the Mini Mental State Examination (MMSE-SR) [14] to ensure that the participants were cognitively fit enough to participate in the study and perform self-monitoring. The MMSE-SR includes 20 items covering eleven areas. The maximum score is 30 and a score < 24 indicates dementia. The 20 participants performed the test with the following results: 28 (*n* = 3), 29 (*n* = 8) and 30 (*n* = 9). One dropped out before the interview due to perceived technical difficulties, resulting in 19 patients being part of the trial. They comprised 12 men and 7 women with a mean age of 59 years (range 27–82 years). The men were older (mean 66 years) than the women (mean 46 years). After the self-monitoring thirteen patients responded to the Being Taken Seriously Questionnaire. The reason for non-response was not analysed.

### Device and patient reported experience measures

#### The Coala Heart Monitor^®^

The Coala Heart Monitor^®^ [15] is a new type of simplified heart monitor for self-monitoring as illustrated by pictures 1 and 2. It is a handheld two-lead ECG recording device with a high sampling rate that in addition to thumb ECGs can also provide chest ECGs. The interpretation is based on pattern recognition algorithms, including POAF detection by means of RR dispersion and atrial signals. The device is connected wirelessly to a smartphone via Bluetooth connectivity. The user can view the interpretation on the smartphone and store PDFs of the tracings in the healthcare provider’s computer. Messages to the patient can also be viewed directly in the app. The Coala Heart Monitor^®^ helps the patient to understand the heart, detect POAF and identify early signs of other heart conditions. The analysis takes 60 s, after which the patient immediately receives the outcome on the smartphone. Every ECG-measurement is digitally stored in the hospital portal and shared with the cardiac surgeon on call and the clinical nurse specialist responsible for follow-up.

**Figure:**
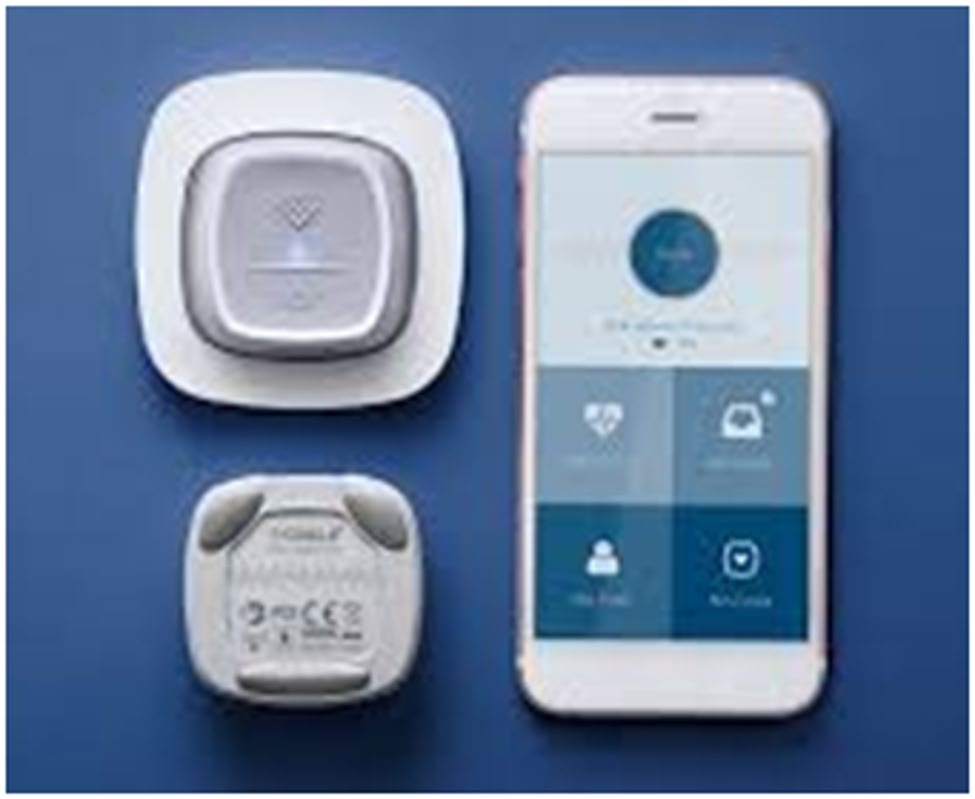
The Coala system and smartphone connection

**Figure:**
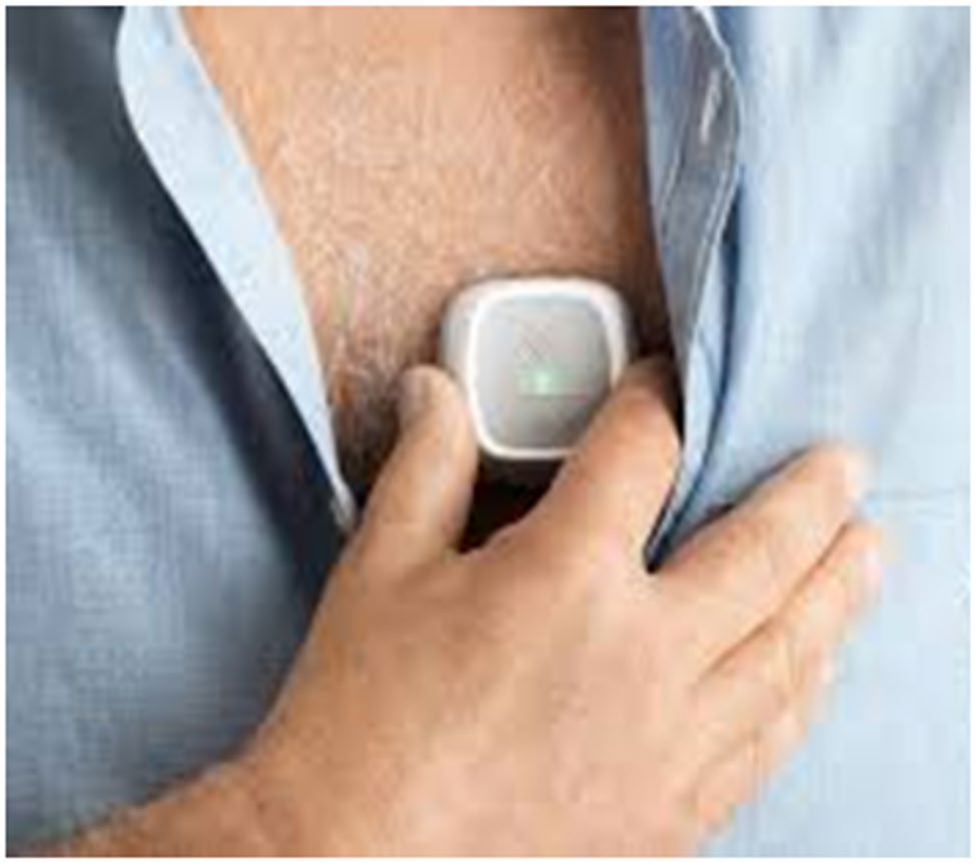
How the Coala Heart Monitor® is used to self-monitor the patient’s heart rhythm

#### Self-monitoring procedure

When discharge was decided, the patients were invited to participate in the trial by two clinical nurse specialists (CNS) in cardiac nursing and written informed consent was obtained. Information and instructions were provided regarding the use of the monitor as well as how data would be transferred to the clinic and stored. They were instructed to perform self-measurements twice daily at 8 am and 4 pm, as well as when suspecting POAF, for four weeks. We tried to detect arrhythmia and symptoms related to possible heart conditions e.g. breathlessness and palpitations. Two CNS were available for questions during the day shift and performed the follow-up of the ECG measurements. In cases of abnormal heart rhythm, the CNS contacted the participants by phone to follow up their condition and provide further instructions based on the cardiac surgeon’s recommendation. Any abnormal rhythm resulted in contact with the patient. Clinical responses were recorded in the patient’s medical record. The nurse contacted the surgeon to validate her assessment and judgement at every event. At the end of the four weeks the participants returned the Coala Heart Monitor^®^ to the clinic in a pre-stamped envelope.

## Data collection

### Semi-structured interviews

After two weeks, a semi-structured telephone interview was performed by the two CNS, and the BTSQ-P questionnaire was completed. The interview primarily focused on five areas of interest, i.e., Could you please tell me about the past two weeks? How did you experience this initial period at home after discharge? How did you experience the use of the Coala as a self-monitoring device? How did you experience your self-management responsibility? How do you communicate and manage your symptoms? How does self-monitoring affect your everyday life? Follow-up questions were posed to clarify the patient’s perspective or deepen understanding. Each participant was interviewed alone and two of the authors took turns performing the interviews based on the principle that the author who had introduced the self-monitoring procedure to the participant also performed the interview to take advantage of the already established trusting relationship. The interviews, which lasted between 30 and 50 min, were digitally recorded and transcribed verbatim. The study was deemed to hold sufficient power to support both depth and variation, supported by the concept of information power in qualitative research [16].

### Secondary variables

Additional variables collected were sex, age, co-morbidities, type of surgery, POAF in the TICU or thoracic ward.

### The being taken seriously questionnaire-patient version (BTSQ-P)

The BTSQ-P was developed in collaboration with patients and based on their responses. It was psychometrically evaluated in high-tech caring environments e.g. cardiac surgical unit [17] and includes the following eight items:

1. The staff listened to me.
2. I received help to understand what has happened.
3. I received help to understand what is about to happen.
4. My concerns have been taken seriously.
5. My symptoms have been taken seriously.
6. I have been taken seriously as a person.
7. The staff made me feel good in the present moment.
8. The staff made me feel safe.

The response can be given on a six-point Likert scale ranging from strongly agree to strongly disagree. The lowest score is 6 and the maximum 48. Inter-item correlations range from 0.759 to 0.908. The internal consistency coefficient measured by Cronbach’s alpha was 0.87 and the ordinal alpha was 0.93. The one factor solution accounts for 80.4% of the variance. The maximum score of 48 means that the patient totally agrees that he or she has been taken seriously in a person-centred manner.

### Ethical issues

Approval was granted by the Swedish Ethical Review Authority (D.nr. 2020–05007) and the ethical requirements outlined in the Declaration of Helsinki were adhered to. The participants gave their written consent.

### Data analysis

We used deductive content analysis in line Hsieh and Shannon [18] guided by the self-management framework developed by Lorig & Holman [19]. This framework includes three parts: medical, role and emotional management. Self-management also involves six different skills, which were explored: problem solving, decision-making, nurse-patient partnership formation, action planning, adjustment and self-responsibility. The interviews were analysed in a stepwise manner: (1) Grasping the framework, (2) Naïve reading of the text, (3) Identifying meaning units in line with the framework, (4) Identifying data not consistent with the self-management framework. During this step an explicit and clear understanding of the meaning of self-monitoring emerged by means of a new category, i.e., the safety bringing device promotes health, illustrated in Fig. 1. (5) In the fifth step, meaning units were condensed. (6) We chose quotations illustrating the findings. (7) The final step involved a comprehensive understanding of the meaning of self-management and self-monitoring after open cardiac surgery. The content analysis was performed by three of the authors separately and in collaboration to compare the analysis and reach consensus.

### Statistics

The statistical analyses were performed using SPSS Statistics (SPSS Inc 25/2020, IBM Corporation, Armonk, N.Y., USA). We explored age differences with Student’s T-test.

### Trustworthiness enhancing techniques

The research group held continuous discussions about rigor and reflexivity, directed by the guide presented by Olmos-Vega, Stalmeijer, Varpio & Kahlke [20]. To ensure reflexivity throughout the data collection, analysis and interpretation the researchers constantly engaged in critical reflection regarding their self-awareness, biases and assumptions about the data.

## Results

### Impact on self-management

The first two research questions were How does self-monitoring with the Coala Heart Monitor^®^ after cardiac surgery affect the patients’ self-management, symptom management, body perception and possible postoperative distress? Is monitoring yourself while awaiting out-patient follow-up a benefit or a burden? The answers will be presented below.

### Medical management

The participants represented two different experiences of being discharged to their home after cardiac surgery, namely health and illness. Those experiencing health lacked distressing symptoms, ate normally and were reasonably physically active in line with their present condition. Participants experiencing illness had to manage more or less typical symptoms. These included feeling breathless, lack of appetite, feeling a throbbing at the back of the head, sternal pain, wound infection in the leg and fluid in the lungs that required hospital admission and thoracic tubes. As reported above, four participants also had arrhythmia and using the Coala became part of symptom management.

“*I felt a lot of stress*,* my heart was beating fast*,* and I didn’t know why actually. It was racing in an awful way…. I felt something happening in my chest. Then I started to measure* [with the device] *and I felt that this is normal*,* I could relax and let go of the stress*” (Female informant, 60 years).

### Role management

Role management involves maintaining or establishing new behaviours or life roles. Cardiac surgery acted as a motivator for changing everyday habits and health behaviour.

*“The only change I need to focus on now is to get these things straight… hyperlipidaemia and first and foremost the diabetes* (Male informant, 62 years).

At the same time, it was challenging to accept that recovery is a time-consuming process involving increased dependency on others due to symptoms and post-surgical restrictions, e.g. not allowed to drive a car for six weeks.

“*I am dependent on my family right now and that is awkward in some situations. I need to get a lift and adjust to my daughter’s schedule to pick up lunch and so on*” (Male informant, 59 years).

Self-monitoring with the device also affected habitual life roles. Older male participants who were used to not paying attention to themselves or talking about feelings experienced positive effects due to feeling safe, receiving confirmation that everything seemed alright, thus confirming their own assessment. The device gave a sense of control to people who did not particularly appreciate losing control. For five male participants over 65 years self-monitoring meant collaborating with a wife or daughter. They performed the monitoring together and shared the experience that everything appeared normal, resulting in a sense of togetherness. No women reported receiving help from relatives.

### Emotional management

Undergoing cardiac surgery meant an existential transition, where the device became a mental support tool. The patients were discharged with a sense of uncertainty, but the device acted as a credible authority and thus self-efficacy for self-management increased. The participants learnt about their bodily signals, drew conclusions on causes and effects and confirmed their conclusions with the monitoring procedure. This enabled a better understanding of their body and heart, which created a sense of security. Two weeks after surgery some participants doubted that they would survive, causing a sense of loneliness due to having experienced something that friends and relatives were unable to relate to. They then contacted the medical social worker for mental support.

“*And then last week*,* after some pressure from my parents*,* siblings and spouse I contacted the medical social worker because I needed someone to talk to. You feel lonely. It is like being in a new world and experiencing things that nobody else knows about*” (Female informant, 34 years).

Some participants feared not waking up again after going to sleep at night and had existential thoughts about how long they will survive and if their condition was hereditary and would be passed on to their children.

“*Of course*,* I have had these thoughts when I go to bed at night. Will I wake up in the morning? It doesn’t last that long though. But it would have been great to get some information about this before surgery*,* that these thoughts might occur*” (Male informant, 56 years).

The oldest participants adopted a fatalistic approach and were solidly anchored in their understanding that most of their life had been lived. The surgery was viewed as an opportunity for a few extra healthy years.

### Problem solving

Problem solving means finding strategies, asking for advice from HCPs and friends, implementing the solution and evaluating the outcome. One example was a participant with thoracic pain who mastered it by making up an explanation for the pain and then relieving it by a combination of different positioning and physical activity. There were also strategies developed to reduce increased heart rate and experienced stress.

“*I could use my knowledge of yoga and meditation to rest in peace and quiet*” (Female informant, 82 years).

Problem solving was also activated when the self-monitoring did not seem to work. A man with professional experience of engineering solved the technical issues, which increased his self-confidence.

### Self-responsibility

Self-responsibility involves how to find resources, e.g. information. None of the participants consulted the internet to manage their symptoms or used other external sources of information. In this study we focused specifically on resource utilization using the device and the interaction with HCPs. All participants perceived the device as easy to use with or without support from significant others.

“*Regardless of what you do today there are these discussions about the technical development*,* digital solutions and how to be more effective and I believe that this [self-monitoring] is a step forward in the healthcare sector to fulfil your obligations*” (Male informant, 59 years).

### Decision-making

A patient’s decision-making is based on access to necessary information to act on changes in one’s health condition. The device comforted both patients and their relatives. The information provided by self-monitoring eliminates the uncertainty about whether the heart is working as it should. There was now a back-up when symptoms occurred that the patient perceived as difficult to manage. When feeling bodily discomfort, the participants consulted the device and became calm and assured that everything was fine. An illustrative example is a male participant who reached an understanding of his arrhythmia and bodily signs through the self-monitoring to the extent that when he developed POAF he called an ambulance and participated in the decision to be left at the scene, i.e. at home, with no fear. He reviewed his measurements and noticed a positive trend, which made him feel safe. On another occasion he did not detect a positive trend and went to the hospital in the ambulance, which also felt good. Thus, self-monitoring supported the decision-making process. Recording the heart rate and drawing conclusions enabled the correct self-care assessment.

“*It doesn’t matter if the heart rate is 52 or 98 if you feel well. If there are no symptoms and your rate is between those two measurements it is no problem. However*,* if you have a heart rate of 98 and you have done three measurements that day showing a rhythm of 70 once and 50 the next time you might want to analyse why the heart rate is so different. When you’re stable at 58 during all three measurements you don’t have to worry at all. Then it is only figures. But I must admit that I try to interpret the different measurements*,* why they are the way they are. Did I have a calm day or was my position different now than the last time I checked?*” (Male informant, 67 years).

### Nurse-patient partnership formation

To establish a solid partnership with HCPs patients must inform them about disease progression or recovery. The participants’ narratives show that the device acts as a bridge between the patient and HCPs at the Thoracic clinic. One aspect was the sense of security involved in being monitored by CNSs and physicians in the Thoracic clinic who contacted the patient in the event of abnormal measurements.

“*I am satisfied that I got the information I needed and felt comfortable contacting the clinic when something was wrong*” (Female informant, 27 years).

Self-monitoring became a way to show gratitude towards the HCPs by returning the positive measurement results, leading to mutual satisfaction within the caring relationship.

“*I wanted you to see the measurement values and think Wow! he appreciates us. It’s about my wish to give you feedback. It shouldn’t be single sided*,* but mutual. My experience is that we have built a nice relationship*,* and the contact has been great*” (Male informant, 67 years).

### Action planning

It is important that the patient develops a realistic action plan including solving technical problems or setting the alarm to remember to take the measurements. One participant synchronized self-monitoring with mealtimes. Action planning also involves organising everyday life to balance physical activity with rest to avoid fatigue.

“*I take my pills at the same time as I check with the device at 8 am. Then I have my breakfast. So*,* there is no difference for me. The measurement point at 4 pm is ok since I need to have some food anyway at that time. So*,* it worked fine between 8 am and 4 pm*” (Male informant, 63 years).

### Adjustment

A person performing self-adjustment uses their skills and previous knowledge to adjust to the current health situation. For the participants it usually meant adjusting daily activities based on the recovery process after cardiac surgery. Rehabilitation raised new demands regarding lifestyle and activities in daily life.

“*This autumn I will reduce my time at work and spend more time with my family*,* finishing my education. That’s what I want. Other things are important. Not necessarily my job but my family and friends*,* being able to take a walk outdoors. I have got a second chance. I am a different person no*w”. (Female informant, 34 years).

### The self-monitoring device is comforting and promotes health

In addition to the self-management framework, the findings revealed that self-monitoring with the device meant feeling safe despite being at a distance from the hospital. None of the participants experienced self-monitoring as a burden. The device was experienced as a companion by an older participant and viewed as a valuable aspect of recovery. The perceived comfort and safety lay in the combination of self-monitoring and follow-up by HCPs. It was easier to monitor one’s heart rate and draw conclusions from it than to understand and manage non-cardiac related symptoms. The device also served as a tool to test physical performance. When pushing oneself a bit harder during an uphill walk and mastering that challenge, the device acted as a confirmation of one’s experienced physical performance.

“*I find it positive when you see that the result is good. Then I feel calmer and more secure. It gives me the courage to take a walk. But if the measurement showed that something was wrong*,* then I would have been scared or more careful or cautious. But now you get confirmation that everything is fine and more positive*” (Male informant, 64 years).

The heart monitor stimulated learning about one’s body and the understanding of one’s symptoms. When there was a discrepancy between the bodily signals and what the monitor showed, most of the participants chose to rely on their own perception of their body rather than the technical device.

“*I want to perform some extra monitoring to understand my body and how it works. What is going on? What happens when I wait for a while and then measure again? It’s like learning to know your body again*,* because something new has occurred. I preferred to continue self-monitoring and even got my own device. Simply to understand how it makes sense now that I have arrhythmia. To have control*”. (Male informant, 56 years).

### Meta-inference

**Fig. 2:**
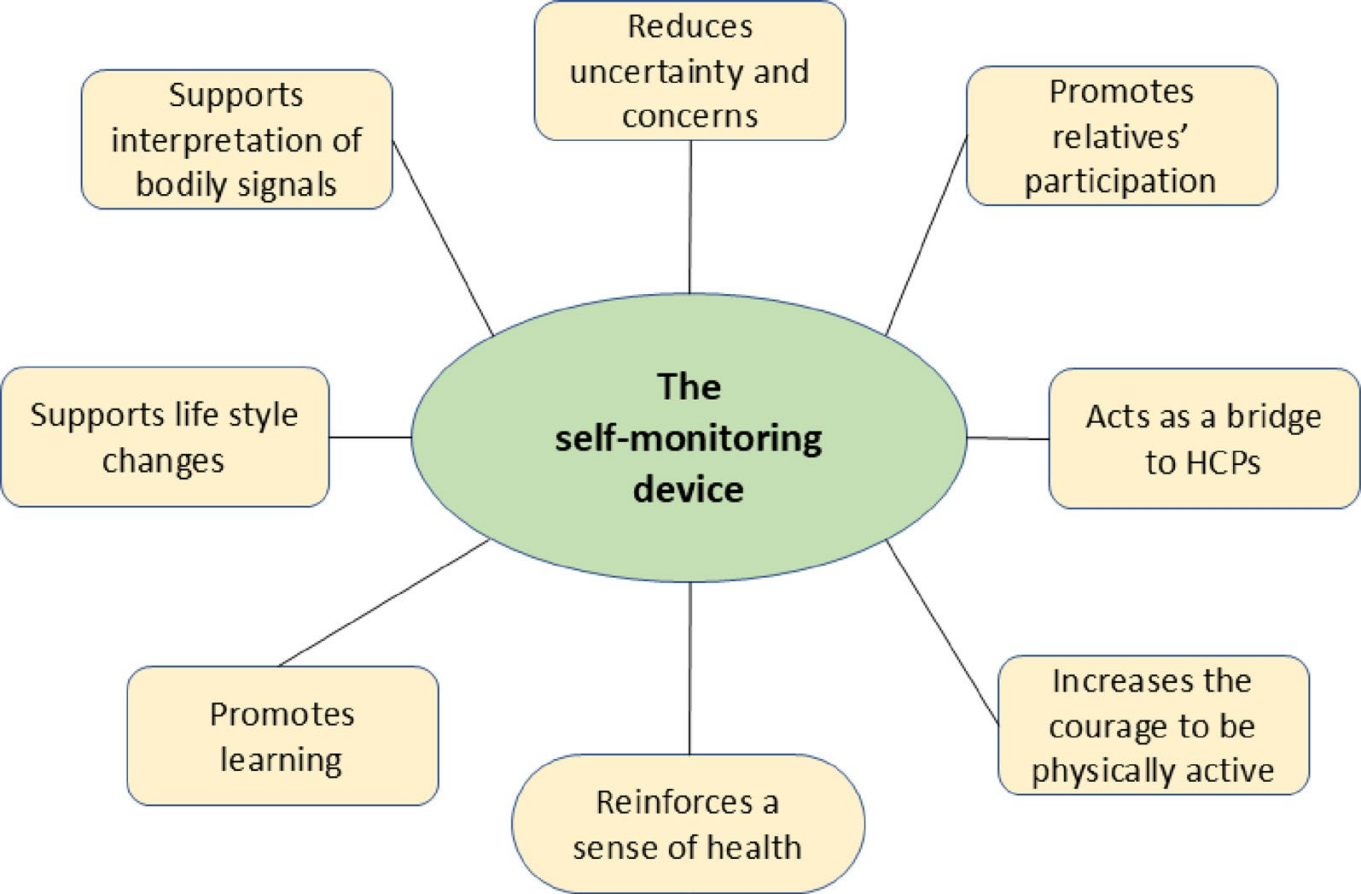
The identified benefits of 19 patients’ self-monitoring of their heart rhythm for a two-week period after discharge from open cardiac surgery

To summarize the benefits of using a self-monitoring device for measuring the heart rate after discharge from cardiac surgery we refer to Fig. 2 and the descriptions above related to each self-management dimension. The quotations illustrate that acceptability of the device was strong. They adhered to the monitoring, and the technical performance of the device was sufficient. At discharge all participants had sinus rhythm. However, four developed arrhythmias during self-monitoring at home. One woman had ventricular extra palpitations in 14 out of 41 measurements. Three men developed POAF. The pattern for these three men was as follows: the first developed persistent POAF after one week of self-monitoring, which remained during the whole self-monitoring trial period. The second had six episodes of POAF during the two-week trial period but had sinus rhythm when the trial ended. Finally, the third man had five episodes of POAF, and his condition deteriorated towards the end of the trial period. Hence, he terminated the trial on his own initiative and was admitted to hospital for treatment. Regardless of POAF or not the participants felt taken seriously as shown in the BTSQ-scores, where the heart monitor acted as a bridge between the HCPs and the participants. Thirteen participants responded to the BTSQ with a score between 41 and 48. The median BTSQ was 47 (p _25_ 45; p_75_ 48) which indicates that the participants felt approached as persons and that the contact between the cardiac unit established via the monitor were built on mutual trust. The monitor increased the participants’ sense of responsibility, will and initiatives, thus promoting a constructive care process (Fig. 2).

## Discussion

The discussion of the findings will focus on the extent to which the primary research questions were answered. In line with Ricoeur’s description [21] of the capable human being, homo capax, we found that the participants were both capable and receptive to learning after discharge from open cardiac surgery. They were able to face challenges during recovery and develop coping strategies. Ambrosio [22] argues that living with a chronic condition is a multidimensional process where five characteristics are developed, i.e., acceptance, coping, self-management, integration and adaptation. We found all these characteristics in our study. The participants used different strategies to develop the skills described by Ambrosio. Open cardiac surgery meant a second chance in life.

Self-monitoring seems to increase and strengthen self-efficacy [23]. Uncertainty decreases when the device confirms the participants’ own judgment. Performance accomplishment is a crucial driver of self-efficacy also shown among heart recipients [24]. A person’s coping behaviour is positively influenced by strong self-efficacy [23]. In our interpretation, when the participants felt capable thanks to the self-monitoring device, their coping abilities were strengthened and their efforts to recover increased further. The monitoring device created a sense of comfort and security, which further stimulated their coping strategies and self-efficacy.

The positive impact of self-monitoring after cardiac surgery is previously described by Lowres et al. [10]. From a nursing perspective the key benefits of this intervention are the sense of comfort, participation, trust in one’s body and symptom management. The discussion about the results of self-monitoring also offered a platform for targeted and person-centred self-care advice from the CNS. For the participants, the personal benefits of self-monitoring are presented in figure two, where in our view one of the most interesting aspects is that self-monitoring triggered a process of learning about one’s body and symptoms. The device provides comfort, but a relevant question is: at what cost? This has not yet been explored, but one assumption is that self-monitoring is cost-effective, as self-care advice based on the measurements can be provided by telephone, thus reducing the number of hospital visits. The Coala^®^ heart monitor was offered to all patients fulfilling the inclusion criteria based on equal access to care. We argue from an ethical perspective that a device that generates comfort, a constructive learning process, enhanced symptom management and stronger caring relationships should be a priority in post operative care after open cardiac surgery.

### Methodological considerations

The participants varied in age from 28 to 83 years, which enabled different perspectives based on life experience and abilities to master the self-monitoring technique. In our county the ratio of men/women undergoing heart surgery is 75/25% and thus this pilot study is considered representative. A strength was that we tested the participants’ cognitive ability for self-monitoring before their participation in the study.

One limitation was that only Swedish speaking patients were included. This was due to the pilot study design, where we wished to test the feasibility of the intervention without the extra work involved in using professional interpreters. Despite limited ethnic diversity, the study contributes an in-depth understanding of self-management in the early stage of recovery after open cardiac surgery and the meaning of using a novel self-monitoring technique. The six-month data collection period took place during the Covid-19 pandemic. This did not affect the recruitment since the operations were scheduled as usual. The data collection procedure was however affected by the restrictions leading to the choice of a telephone interview. The study participants would not have been contacted at all regardless of the pandemic or not. Thus, they got more attention by participating in the study, not experiencing the same abandonment as other patients during the pandemic restrictions.

The participants performed measurements during the first two post-operative weeks starting immediately after discharge. In the event of a large-scale study, the measurement period will be prolonged to four weeks based on the participants’ desire for a longer duration of self-monitoring. The dual role of the CNSs both implementers of the intervention and interviewers may have influenced patient responses. Our impression was that the trusting relationship between the implementers and the patients enriched the patients’ narratives about their situation after discharge. They felt safe and thus became more authentic when elaborating on their experiences. However, there is of course an inherent bias when implementers also collect the data that shouldn’t be neglected.

The semi-structured interviews were analysed using the framework developed by Lorig & Holman, which was helpful for identifying meaning units. All researchers discussed the data to increase credibility.

Some technical issues occurred during the study both with the heart monitor and the application recording the heart rate. The main challenge was that fake social identification numbers were created in the application to ensure the study participants’ anonymity. This problem was solved by out-of-the-box solutions from the company providing the monitor. One patient declined participation due to postoperative fatigue and one due to unwillingness to use apps. One was forced to end the trial because the self-monitoring did not seem to function, despite changes to the device. The ongoing pandemic and heavy pressure on the clinic did not lead to any errors or drop-out, which is an aspect in favour of the procedure. Since the study is performed in Scandinavia the transferability concerns primarily Scandinavian health care systems or European countries with similar discharge routines and postoperative support structures.

## Conclusion

We conclude that POAF after open cardiac surgery causes concern, a degree of suffering and a sense of vulnerability that can be reduced or relieved by self-monitoring of one’s heart rate, thus providing a sense of security despite distance from the hospital. Self-monitoring stimulates learning about one’s body and symptoms in a highly useful way that can be adopted by both young and older patients, in addition to promoting relatives’ constructive participation.

### Clinical implications

The extensive advantages identified by this pilot feasibility study suggest large-scale implementation of self-monitoring in the first four weeks after discharge from open cardiac surgery and an evaluation of cost effectiveness. The self-monitoring should and could easily be supervised by a CNS in cardiac nursing in collaboration with the thoracic surgeon on call.

## Data Availability

Raw data in Swedish are available on request.
